# The impact of dietary and lifestyle factors on obesity among chinese children during the first 6 years of life: a 2014-2015 multi-centre study

**DOI:** 10.3389/fnut.2026.1854091

**Published:** 2026-09-10

**Authors:** Yan Zhao, Wen Zheng, Yufei Ni, Aiping Wu, Kan Ye, Yelin Bao, Xinye Jiang, Guoqin Liu, Guoqiang Yang, Li Zhang, Hongxia Qi, Heyun Lv, Lihua Li, Rui Qin, Hong Cao

**Affiliations:** 1 Department of Nutrition, Affiliated Hospital of Jiangnan University, Wuxi, Jiangsu, China; 2 Wuxi School of Medicine, Jiangnan University, Wuxi, China; 3 Department of Clinical Nutrition, Jiangsu Province Hospital, the First Affiliated Hospital With Nanjing Medical University, Nanjing, Jiangsu, China; 4 Institute of Future Food Technology, JITRI, Yixing, China; 5 Department of Child Health Care, Yancheng Maternity and Child Health Care Institute, Yancheng, China; 6 Department of Child Health Care, Nantong Maternity and Child Health Care Hospital, Nantong, China; 7 Department of Child Health Care, Xinghua Maternity and Child Health Care Hospital, Xinghua, China; 8 Department of Child Health Care, Suzhou Municipal Hospital, Suzhou, China; 9 Department of Child Health Care, Drum Tower Maternity and Child Health Care Institute, Nanjing, China; 10 Department of Child Health Care, Wuxi Maternity and Child Health Care Hospital, Wuxi, China; 11 Department of Child Health Care, Dafeng Maternity and Child Health Care Hospital, Dafeng, China; 12 Department of Child Health Care, Kunshan Maternity and Child Health Care Institute, Kunshan, China; 13 Department of Child Health Care, Huai’an Maternity and Child Health Care Hospital, Huai’an, China; 14 Department of Child Health Care, Xuzhou Children’s Hospital, Xuzhou, China; 15 Department of Child Health Care, Jiangning Maternity and Child Health Care Institute, Nanjing, China; 16 Department of Clinical Nutrition, Lianshui People’s Hospital of Kangda College Affiliated to Nanjing Medical University, Huai’an, Jiangsu, China; 17 Jiangsu Women and Children Health Hospital, Women and Child Branch Hospital of Jiangsu Province Hospital, the First Affiliated Hospital With Nanjing Medical University, Nanjing, Jiangsu, China

**Keywords:** children, dietary intake, lifestyle, multi-centre study, obesity, physical activity, sleep duration

## Abstract

**Objective:**

To identify lifestyle and dietary factors associated with obesity in children during the first 6 years of life.

**Methods:**

Children aged 0–6 years were recruited from 12 Children’s Health Care Centers in Jiangsu, China, by a multistage stratified cluster random-sampling method. Diet, sleep, supplementation and outdoor activity were collected by questionnaire/interview. Obesity was defined as Body mass index Z-score >2 (World Health Organization standards). Logistic regression was used to examine the associations between lifestyle/dietary factors and obesity, adjusting for sex, age, delivery mode, birth weight, region, and season, with additional stratified and restricted cubic spline dose-response analyses.

**Results:**

A survey of 5,243 children were enrolled in the present study. Prevalence varied by characteristics: sleep <10 h (8.5%), >14 h (9.9%), outdoor activity <1 h (10.7%), complementary feeding before 4 months (9.8%), daily calcium ≥300 mg (10.4%), preference for meat (10.0%), fish/shrimp ≥ 2 times/week (7.1%), daily milk ≥600 mL (6.3%), daily meat >150 g (11.2%), and daily egg >60 g (11.1%). Adjusted analyses identified higher obesity odds for sleep <10 h [odds ratio (OR) 1.327, 95% Confidence Interval (95% CI) 1.024–1.719] or >14 h (2.503, 1.640–3.820), outdoor activity <1 h (2.018, 1.465–2.779), vitamin D supplementation <15 days (1.686, 1.063–2.673), complementary feeding <4 months (1.665, 1.114–2.487), preference for meat (1.369, 1.049–1.859), fish/shrimp ≥ 2 times/week (1.354, 1.026-1.788), daily meat >150 g (2.060, 1.207-3.514), daily egg >60 g (2.937, 1.075–8.024), and daily milk ≥600 mL (1.830, 1.083–3.094 vs. ≤100 mL). A vegetarian preference was inversely associated with obesity.

**Conclusion:**

There was a non-linear association between obesity and sleep duration. Lack of outdoor activity was an independent associated factor for obesity. In nominal analyses, excessive intake of milk, fish/shrimp, meat, and eggs was associated with higher odds of obesity in children during the first 6 years of life, whereas an inverse association was observed, with obesity risk decreasing among individuals who preferred vegetarian foods. After false discovery rate correction, sleep duration >14 h, outdoor activity <1 h/day, preference for vegetarian foods, and meat intake >150 g/day remained statistically significant.

## Introduction

Children living with obesity may raise risks of asthma, sleep apnoea, cardiovascular disease (CVD), and type 2 diabetes in childhood (1), and adult obesity, stroke, cancer, and other chronic diseases long-term (2). The etiology of early childhood obesity was multi-factorial, including behavioral, genetic, physical activity (PA), duration, and environmental determinants.

However, the specific contribution of sleep duration, particularly in the critical 0–6-year age group, remained a subject of ongoing debate, with a paucity of studies targeting this demographic. PA, defined as any bodily movement produced by skeletal muscles that results in energy expenditure, was inversely correlated with adipose-related factors in preschoolers, with sex-specific differences in PA patterns emerging with age (3). Evidence suggests that vitamin D deficiency may be associated with pediatric obesity (4), though supplementation has not consistently demonstrated efficacy in weight reduction (5). While calcium was integral to energy metabolism, the relationships between vitamin D, calcium supplementation, and adiposity in young children were not well-defined, highlighting the need for larger-scale longitudinal investigations.

Childhood represented a formative period for establishing dietary patterns and eating behaviors. While numerous studies had examined broad dietary patterns in relation to overweight and obesity (6, 7), there is limited research on the specific associations between daily intake of core food groups (e.g., meat, fish, eggs, milk) and obesity prevalence in young children.

In China, national data indicate an obesity prevalence of 3.6% among children under 6 years (8). Regional studies, such as one in Xiamen, reported an obesity prevalence of 5.66% in 56,738 children aged 2–7 years (9). Despite this, comprehensive epidemiological data for children aged 0–6 years in Jiangsu Province were scarce (10). To facilitate early identification and targeted intervention, it was crucial to characterize the demographic and modifiable lifestyle factors - including diet, outdoor activity, and micronutrient supplementation-associated with adiposity in this population. This study therefore aims to investigate the associations between the prevalence of obesity and sleep duration, outdoor activity, vitamin D and calcium supplementation, and daily dietary intake among children aged 0–6 years in Jiangsu Province, China.

## Methods

### Participants and trial design

**FIGURE 1:**
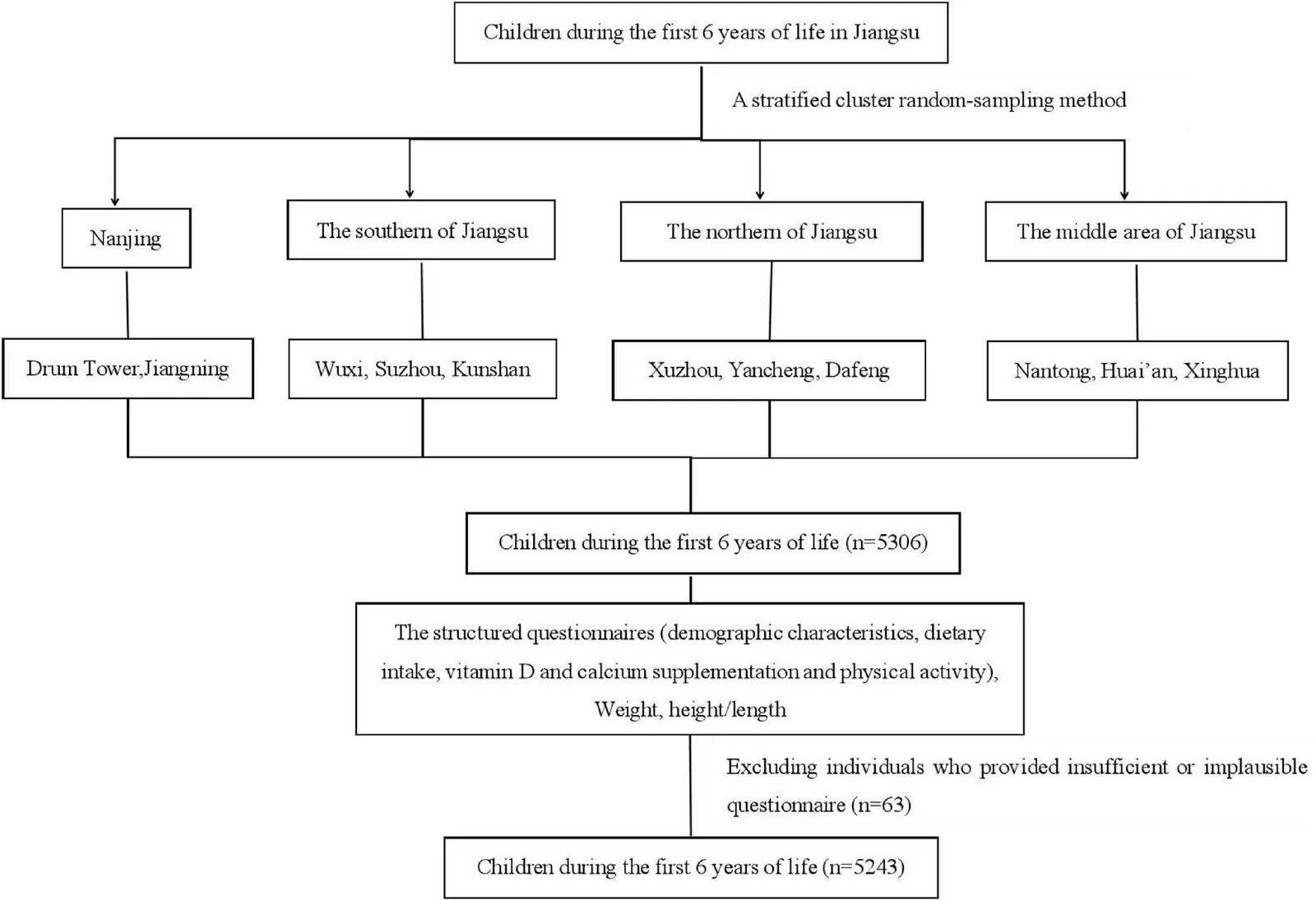
Flowchart of the study.

This investigation constituted a large, population-based, multi-center cross-sectional study. All exposure information, including dietary intake, sleep, and activity behaviors, was collected via retrospective questionnaires, in which caregivers were asked to recall children’s lifestyle and dietary conditions over the preceding period at the single cross-sectional survey time point. Participants were children aged 0–6 years recruited from ten cities across Jiangsu Province between April 2014 and March 2015. In this study, Jiangsu province stratified into four geographical districts, from which three to four children’s healthcare centers were randomly selected per district for participant recruitment (Figure 1). Detailed exclusion/inclusion criteria and the sampling methodology have been previously published (11). Trained interviewers conducted face-to-face interviews with parents or caregivers using structured questionnaires. Standardized anthropometric measurements (weight and height/length) were obtained for all children. The study protocol was approved by the Institutional Review Board of the First Affiliated Hospital of Nanjing Medical University (2014-SR-167). All procedures conformed to the ethical principles of the Declaration of Helsinki, and written informed consent was obtained from all participants.

### Assessment of demographic characteristics

Demographic data, including the child’s gender, age, birth weight, gestational age, mode of delivery, region of residence, season of survey, etc. were collected via the structured questionnaire.

### Assessment of dietary Intake, Supplementation, and outdoor activity

Within this cross-sectional survey, we utilized retrospective self-reported questionnaires to collect historical behavioral and dietary data.

Dietary intake over the preceding 3 months was assessed using a validated, semi-quantitative food frequency questionnaire (FFQ) comprising 59 food items across 14 food groups (12), complemented by a photographic atlas for portion size estimation (13). Dietary preferences were categorized as: no preference, preference for sweets, preference for meat, or preference for vegetarian foods.

Information on vitamin D and calcium supplementation was collected, including timing of initiation post-birth, brand, dosage, and formulation. Any supplement containing vitamin D or calcium was recorded as such in the previous paper (14).

Outdoor activity levels were assessed using a validated questionnaire (15), quantifying the typical daily duration (hours per day) of outdoor activity.

### Assessments of sleep duration

Average total sleep duration (nighttime sleep plus afternoon nap) over the prior month was estimated using age-appropriate, validated instruments: the Brief Infant Sleep Questionnaire (BISQ) (16) for children aged 0–2 years and the Children’s Sleep Habits Questionnaire (CSHQ) (17) for children aged 3–6 years.

1. Nighttime sleep duration: period from nighttime bedtime to morning wake-up time, with awakenings during the night excluded.

2. Afternoon nap duration.

The “average sleep time” for each child was calculated as the sum of their nighttime sleep duration and afternoon nap duration.

### Anthropometric measurements and definitions

Weight and recumbent length (0–24 months) or standing height (25–71 months) were measured using calibrated equipment following standard protocols (18). The Body Mass Index Z-score (ZBMI) was calculated based on World Health Organization (WHO) growth standards (19). Obesity was defined as ZBMI > +2 SD (19).

### Stratified grouping criteria for sleep Duration, outdoor Activity, and daily food consumption

Daily intake (milk, meat, and other food items) were converted to continuous grams or milliliters., sleep duration and outdoor activity were treated as continuous variables in time-based units (e.g., hours per day). For stratified regression analyses, categorical cut-off points for each food group (milk: ≤100 mL, 100–600 mL, ≥600 mL; meat: <50 g, 50–150 g, >150 g) were determined according to the distribution of intake data in the present study population. All cutoffs were defined using percentiles of the continuous consumption data, with additional consideration of real-world dietary patterns among preschool children to ensure subgroup balance.

### Selection of covariates

Before constructing the multi-variable regression model, all candidate predictors underwent univariate screening. Consistent with our prior investigations (20), child sex, age, mode of delivery, birth weight, region, and season of investigation demonstrated significant associations with the prevalence of childhood obesity. Accordingly, these variables were designated as core covariates.

### Statistical analysis

Participant characteristics were presented as frequencies and percentages. Chi-square tests were used for categorical variable comparisons. Age-specific associations were examined by further stratifying Chi-square analyses across the following age groups: 0–6, 7–23, and 24–71 months. Unadjusted and adjusted associations between relevant factors and obesity prevalence were evaluated using logistic regression, calculating odds ratios (ORs) with 95% confidence intervals (CIs). Stratified analyses were conducted for specific dietary variables by age group. All analyses were performed using SPSS version 26.0, with a two-sided *P*-value < 0.05 considered statistically significant.

### Restricted cubic spline (RCS) analysis

In this study, sleep duration was initially ascertained as an interval-categorical variable. To examine potential nonlinear dose-response associations between sleep duration and childhood obesity risk, the categorical sleep measure was operationalized as an approximate continuous variable and subsequently analyzed using a restricted cubic spline (RCS) model. Following the methodology of Jiawei Yin et al. (21), variable assignment was defined as follows: for categories with explicit upper and lower bounds, the midpoint of each interval served as the representative value. For the two open-ended extreme categories lacking defined boundaries, values were approximated based on the interval width of adjacent groups-7 h was assigned to the category with sleep duration less than 8 h, and 17 h to that exceeding 16 h. Given the limited sample size within the extreme sleep duration groups, three knots were prespecified at 9, 11, and 15 h (corresponding to the 5th, 50th, and 95th percentiles of the distribution). All RCS models were fully adjusted for confounding covariates, including child sex, age, mode of delivery, birth weight, study region, and investigation season. The overall and nonlinear associations between sleep duration and childhood obesity risk were statistically evaluated using the Wald χ^2^ test and nonlinearity was assessed by testing the joint significance of the nonlinear spline terms via the Wald χ^2^ test. All RCS analyses were performed using R (version 4.6.0) with the “rms” package.

### False discovery rate (FDR) analysis

To avoid type I error inflation caused by multiple statistical tests, the False Discovery Rate (FDR) method was used for multiple testing correction of all original *P*-values. The FDR-adjusted *P*-values of the unadjusted logistic regression (P1_FDR) and the adjusted logistic regression (P2_FDR) were obtained respectively. All statistical tests were two-tailed, and a corrected *P*-value < 0.05 was defined as statistically significant. FDR correction was performed using R (version 4.6.0) to ensure the accuracy and reliability of the results.

## Results

A total of 5,243 children were enrolled in the present study (Table 1). Significantly higher prevalence was observed in subgroups with: sleep duration <10 h (8.5%), sleep duration >14 h (9.9%), daily outdoor activity <1 h (10.7%), introduction of complementary feeding before 4 months (9.8%), a stated preference for meat (10.%), milk daily intake ≥600 mL (6.3%), meat daily intake >150 g (11.2%), and egg daily intake >60 g (11.1%).

**TABLE 1:** The comparison of the prevalence of obesity among children during the first 6 years of life.

Variables	N	ZBMI > 2	ZBMI ≤ 2
		N (%)	N (%)
Daily sleep duration for children	5,243	353 (6.7%) **	4,890 (93.3%)
<10 h	1,565	133 (8.5 %)	1,432 (91.5%)
10–14 h	3,335	186 (5.6 %)	3,149 (94.4%)
>14 h	343	34 (9.9 %)	309 (90.1 %)
Time of outdoor activity every day for children	5,139	341 (6.6%) **	4,798 (93.4%)
<1 h	505	54 (10.7%)	451 (89.3%)
≥1 h	4,634	287 (6.2%)	4,347 (93.8%)
Feeding patterns for infants from birth to 6 months	5,238	353 (6.7%)	4,885 (93.3%)
Breastfeeding	2,700	176 (6.5 %)	2,524 (93.5 %)
Mixed feeding	1,515	105 (6.9 %)	1,410 (93.1 %)
Artificial feeding	1,023	72 (7.0 %)	951 (93.0 %)
Vitamin D supplementation for infants from birth to 6 months	5,068	324 (6.4%) *	4,744 (93.6%)
Yes	3,725	223 (6.0%)	3,502 (94.0%)
No	1,343	101 (7.5%)	1,242 (92.5%)
The initial time of vitamin D supplementation after birth	3,584	214 (6.0%)	3,370 (94.0%)
<15 days	351	28 (8.0%)	323 (92.0%)
15–42 days	1,384	69 (5.0%)	1,315 (95.0%)
>42 days	1,849	117 (6.3%)	1,732 (93.7%)
The initial time of complementary feeding	4,848	313 (6.5%) *	4,535 (93.5%)
<4 months	306	30 (9.8%)	276 (90.2%)
≥4 months	4,542	283 (6.2%)	4,259 (93.8%)
Vitamin D supplementation for children in the last 3 months	5,198	349 (6.7%)	4,849 (93.3%)
Yes	1,476	102 (6.9%)	1,374 (93.1%)
No	3,722	247 (6.6%)	3,475 (93.4%)
Daily dose of vitamin D supplementation for children in the last 3 months	1,303	83 (6.4%)	1,220 (93.6%)
<400 IU	898	57 (6.3%)	841 (93.7%)
≥400 IU	405	26 (6.4%)	379 (93.6%)
Calcium supplementation for children in the last 3 months	5,151	342 (6.6%)	4,809 (93.4%)
Yes	985	61 (6.2%)	924 (93.8%)
No	4,166	281 (6.7%)	3,885 (93.3%)
Daily dose of calcium supplementation for children	782	42 (5.4%)	740 (94.6%)
≤300 mg	734	37 (5.0%)	697 (95.0%)
>300 mg	48	5 (10.4%)	43 (89.6%)
Dietary habit	4,194	271 (6.5%) **	3,923 (93.5%)
No preference	2,534	156 (6.2%)	2,378 (93.8%)
Preference for meat	887	89 (10.0%)	798 (90.0%)
Preference for vegetarian foods	342	6 (1.8%)	336 (98.2%)
Preference for sweets	431	20 (4.6%)	411 (95.4%)
Frequency of fish/shrimp intake	4,336	280 (6.5%) *	4,056 (93.5%)
<2 times/week	1,488	79 (5.3%)	1,409 (94.7%)
≥2 times /week	2,848	201 (7.1%)	2,647 (92.9%)
Milk daily intake for children	4,492	268 (6.0%)	4,224 (94.0%)
≤100 mL	839	46 (5.5%)	793 (94.5%)
100–600 mL	3,130	189 (6.0%)	2,941 (94.0%)
≥600 mL	523	33 (6.3%)	490 (93.7%)
Meat daily intake for children	4,023	261 (6.5%) *	3,762 (93.5%)
<50 g	3,008	188 (6.3%)	2,820 (93.7%)
50–150 g	818	51 (6.2%)	767 (93.8%)
>150 g	197	22 (11.2%)	175 (88.8%)
Egg daily intake for children	4,216	270 (6.4%) **	3,946 (93.6%)
0 g	157	5 (3.2%)	152 (96.8%)
0–60 g	3,851	242 (6.3%)	3,609 (93.7%)
>60 g	208	23 (11.1%)	185 (88.9%)

*P < 0.05,

**P < 0.01

Table 2 presents age-stratified associations between lifestyle/dietary factors and childhood obesity across age groups of 0–6, 7–23 and 24–71 months. Sleep duration showed no significant correlation in the two younger groups but was significant at 24–71 months. Outdoor activity time was significantly associated across all age groups (*P* < 0.05). Fish/shrimp intake was significant at 7–23 months and 24–71 months (*P* < 0.05), but not in infants aged 0–6 months. Daily milk intake showed no significant association in any group. Daily meat intake was not significant in younger groups but significant at 24–71 months (*P* < 0.05). Daily egg intake was not significant at 0–6 or 7–23 months, but significant at 24–71 months (*P* < 0.05).

**TABLE 2:** Age-stratified associations between daily lifestyle and dietary factors and childhood obesity.

Variables	N	ZBMI > 2	ZBMI ≤ 2
		N (%)	N (%)
Daily sleep duration for children			
0–6 months	576	44 (7.6%)	532 (92.4%)
<16 h	555	40 (7.2%)	515 (92.8%)
>16 h	21	4 (19.0%)	17 (81.0%)
7–23 months	1,282	70 (5.5%)	1,212 (94.5%)
<14 h	1,174	61 (5.2%)	1,113(94.8%)
14–16 h	103	8 (7.8%)	95 (92.2%)
>16 h	5	1 (20.0%)	4 (80.0%)
24–71 months	3,385	239 (7.1%) **	3,146 (92.9%)
<8 h	37	2 (5.4%)	35 (94.6%)
8–10 h	1,365	124 (9.1%)	1,241 (90.9%)
10–14 h	1,958	109 (5.6%)	1,849 (94.4%)
>14 h	25	4 (16.0%)	21 (84.0%)
Time of outdoor activity every day for children			
0–6 months	563	43 (7.6%) **	520 (92.4%)
<1 h	190	20 (10.5%)	170 (89.5%)
1–3 h	348	18 (5.2%)	330 (94.8%)
≥4 h	25	5 (20.0%)	20 (80.0%)
7–23 months	1,265	69 (5.5%) **	1,196 (94.5%)
<1 h	100	14 (14.0%)	86 (86.0%)
1–2 h	534	31 (5.8%)	503 (94.2%)
≥3 h	631	24 (3.8%)	607 (96.2%)
24–71 months	3,311	229 (6.9%) *	3,082 (93.1%)
<1 h	215	20 (9.3%)	195 (90.7%)
1–3 h	2,687	192 (7.1%)	2,495 (92.9%)
≥4 h	409	17 (4.2%)	392 (95.8%)
Frequency of fish/shrimp intake			
0–6 months	56	5 (8.9%)	51 (91.1%)
≤2 times/week	52	4 (7.7%)	48 (92.3%)
>2 times /week	4	1 (25.0%)	3 (75.0%)
7–23 months	1,043	53 (5.1%) *	990 (94.9%)
≤2 times/week	646	24 (3.7%)	622 (96.3%)
>2 times /week	397	29 (7.3%)	368 (92.7%)
24–71 months	3,237	222 (6.9%) *	3,015 (93.1%)
≤1 times/week	985	54 (5.5%)	931 (94.5%)
>1 times /week	2,252	168 (7.5%)	2,084 (92.5%)
Milk daily intake for children			
0–6 months	308	22 (7.1%)	286 (92.9%)
<300 mL	84	4 (4.8%)	80 (95.2%)
≥300 mL	224	18 (8.0%)	206 (92.0%)
7–23 months	1,096	56 (5.1%)	1,040 (94.6%)
≤100 mL	98	2 (2.0%)	96 (98.0%)
100–800 mL	966	52 (5.4%)	914 (94.6%)
>800 mL	32	2 (6.3%)	30 (93.7%)
24–71 months	3,088	190 (6.2%)	2,898 (93.8%)
0 mL	462	22 (4.8%)	440 (95.2%)
0–600 mL	2,585	164 (6.3%)	2,421 (93.7%)
>600 mL	41	4 (9.8%)	37 (90.2%)
Meat daily intake for children			
0–6 months	56	4 (7.1%)	52 (92.9%)
0 g	45	4 (8.9%)	41 (91.1%)
>0 g	11	0 (0.0%)	11 (100%)
7–23 months	931	47 (5.0%)	884 (95.0%)
<100 g	898	45 (5.0%)	853 (95.0%)
≥100 g	33	2 (6.1%)	31 (93.9%)
24–71 months	3,036	210 (6.9%)*	2,826 (93.1%)
≤50 g	2,452	158 (6.4%)	2,294 (93.6%)
50–100 g	339	25 (7.4%)	314 (92.6%)
>100 g	245	27 (11.0%)	218 (89.0%)
Egg daily intake for children			
0–6 months	101	6 (5.9%)	95(94.1%)
≤10 g	60	3 (5.0%)	57 (95.0%)
>10 g	41	3 (7.3%)	38 (92.7%)
7–23 months	1,068	58 (5.4%)	1,010 (94.6%)
≤10 g	110	2 (1.8%)	108 (98.2%)
10–20 g	109	5 (4.6%)	104 (95.4%)
>20 g	849	51 (6.0%)	798 (94.0%)
24–71 months	3,047	206 (6.8%)**	2,841 (93.2%)
<100 g	2,975	195 (6.6%)	2,780 (93.4%)
≥100 g	72	11 (15.3%)	61 (84.7%)

*P < 0.05,

**P < 0.01

**FIGURE 2:**
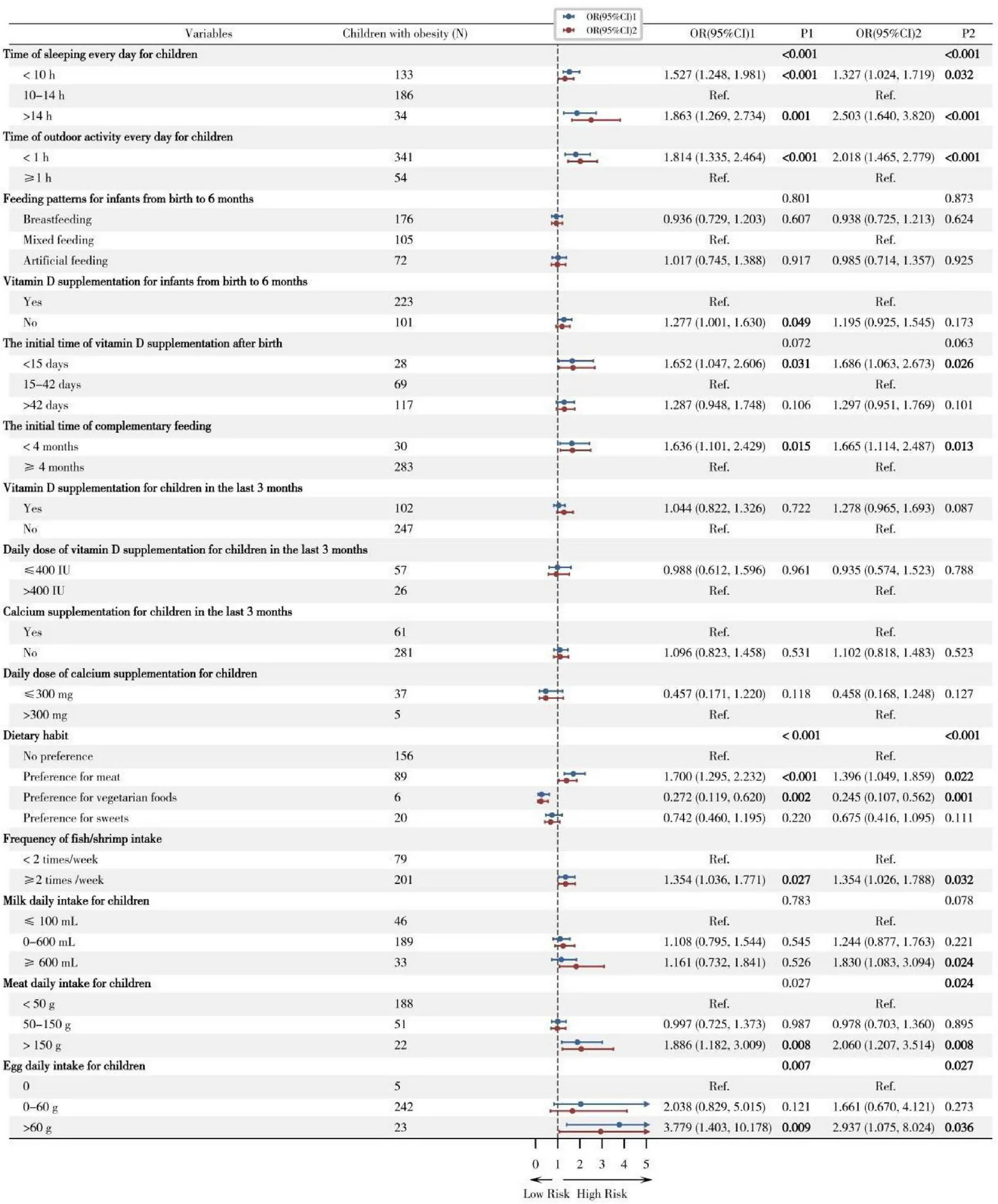
Relationship between lifestyles and dietary factors and obesity in children during the first 6 years of life. P2: Adjusted for sex, age, mode of delivery, birth weight, region and season of investigation.

Figure 2 illustrated odds ratios (ORs) [95% confidence intervals (CIs)] for obesity associated factors before and after adjustment. Compared with reference groups (sleep duration 10-14 h, outdoor activity ≥1 h, got vitamin D supplementation from birth to 6 months, vitamin D supplementation 15–42 days after birth, complementary feeding started ≥4 months, no dietary preference, fish/shrimp intake < 2 times/week, meat daily intake < 50 g, daily egg intake = 0 g), higher obesity association was found in: sleep duration <10 h [OR (95% CI) = 1.527 (1.248, 1.981)], sleep duration >14 h [OR (95% CI) = 1.863 (1.269, 2.734)], daily outdoor activity <1 h [OR (95% CI) = 1.814 (1.335, 2.464)], absence of vitamin D supplementation from birth to 6 months [OR (95% CI) = 1.277 (1.001, 1.630)], vitamin D supplementation <15 days after birth [OR (95% CI) = 1.652 (1.047, 2.606)], complementary feeding started <4 months [OR (95% CI) = 1.636 (1.101, 2.429)], preference for meat [OR (95% CI) = 1.700 (1.295, 2.232)], fish/shrimp intake ≥2 times/week [OR (95% CI) = 1.354 (1.036, 1.771)], meat daily intake >150 g [OR (95% CI) = 1.886 (1.182, 3.009)], and egg daily intake >60 g [OR (95% CI) = 3.779 (1.403, 10.178)] (all *P* < 0.05). Among these, the associations for absence of vitamin D supplementation from birth to 6 months, vitamin D supplementation <15 days after birth, and fish/shrimp intake ≥2 times/week were only nominally significant and was considered exploratory. In contrast, a preference for vegetarian foods was associated with a markedly lower risk [OR (95% CI) = 0.272 (0.119, 0.620)] versus no dietary preference.

Following adjustment for confounders (sex, age, mode of delivery, birth weight, region, and season of investigation), the following associations remained significant (Figure 2): sleep duration <10 h [OR (95% CI) = 1.327 (1.024, 1.719)], sleep duration >14 h [OR (95% CI) = 2.503 (1.640, 3.820)], and daily outdoor activity <1 h [OR (95% CI) = 2.018 (1.465, 2.779)], vitamin D supplementation <15 days after birth [OR (95% CI) = 1.686 (1.063, 2.673)], complementary feeding started <4 months [OR (95% CI) = 1.665 (1.114, 2.487)], preference for meat [OR (95% CI) = 1.369 (1.049, 1.859)], fish/shrimp intake ≥2 times/week [OR (95% CI) = 1.354 (1.026, 1.788)], meat daily intake >150 g [OR (95% CI) = 2.060 (1.207, 3.514)], and egg daily intake >60 g [OR (95% CI) = 2.937 (1.075, 8.024)]. Among the covariate-adjusted associations, those with a lower bound of the 95% CI close to 1.000 should be considered exploratory. These included sleep duration <10 h, vitamin D supplementation supplementation <15 days after birth, preference for meat, fish/shrimp intake ≥2 times per week, and daily egg intake >60 g. Interesting was that among children whose daily milk intake reached ≥600 mL, the adjusted odds ratio was 1.830 (95% CI: 1.083, 3.094), reflecting a nominally significant elevation in the odds of obesity relative to the ≤100 mL reference group, this finding was also exploratory. In contrast, a preference for vegetarian foods was associated with a markedly lower risk [OR (95% CI) = 0.245 (0.107, 0.562)] versus no dietary preference.

**FIGURE 3:**
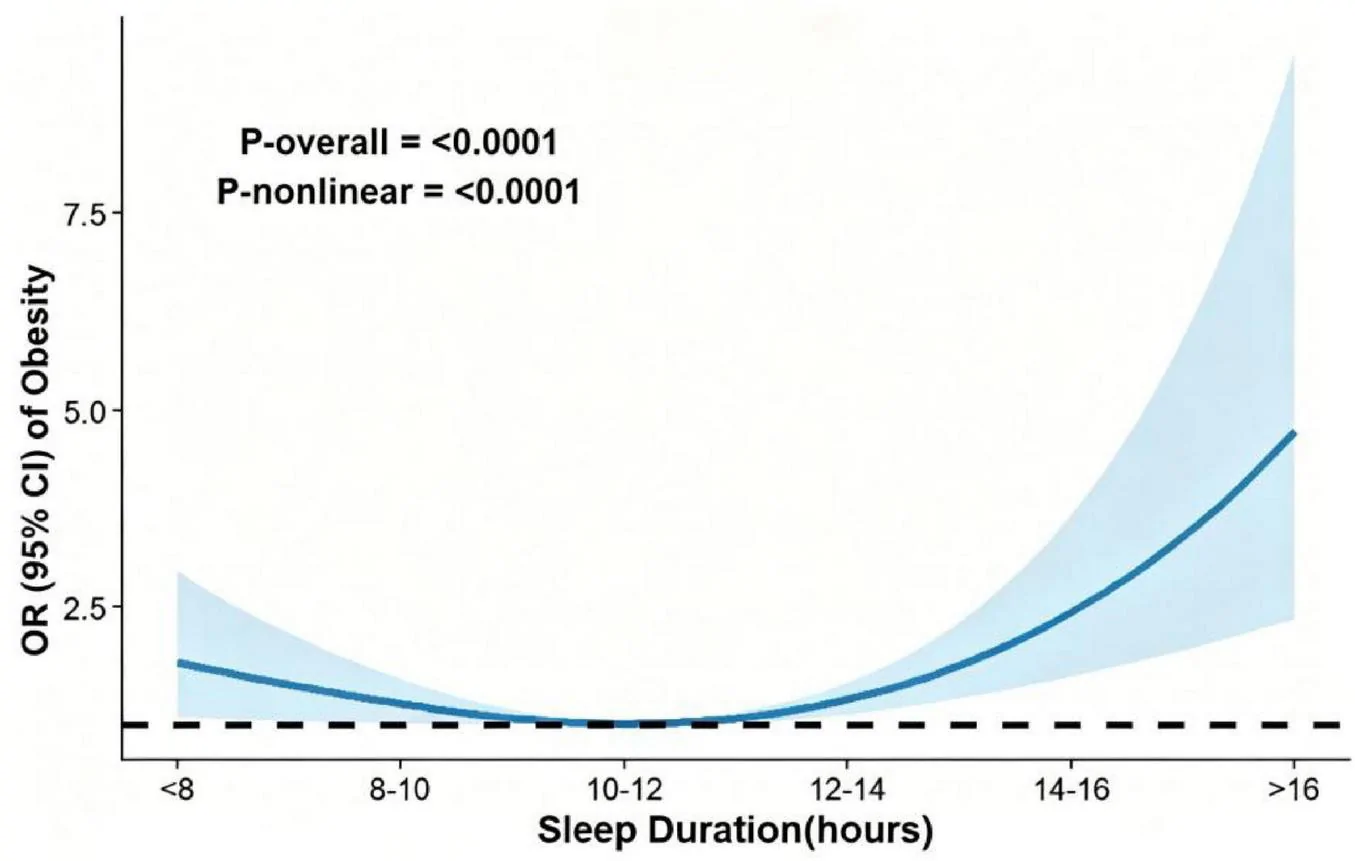
Association between sleep duration and odds ratio (OR) of childhood obesity after adjustment. *Adjusted for sex, age, mode of delivery, birth weight, region and season of investigation.

After adjusting for covariates including child sex, age, mode of delivery, birth weight, region, and season of investigation, the RCS analysis (Figure 3) revealed a nonlinear association between sleep duration and childhood obesity risk, with lower obesity risk observed around 10–12 h of sleep. The global association test indicated a significant relationship between sleep duration and childhood obesity (Wald χ^2^ = 19.68, df = 2, *P* < 0.001); the test for non-linearity was also statistically significant (Wald χ^2^ = 16.23, df = 1, *P* < 0.001), suggesting that the association is not a simple linear one.

**FIGURE 4:**
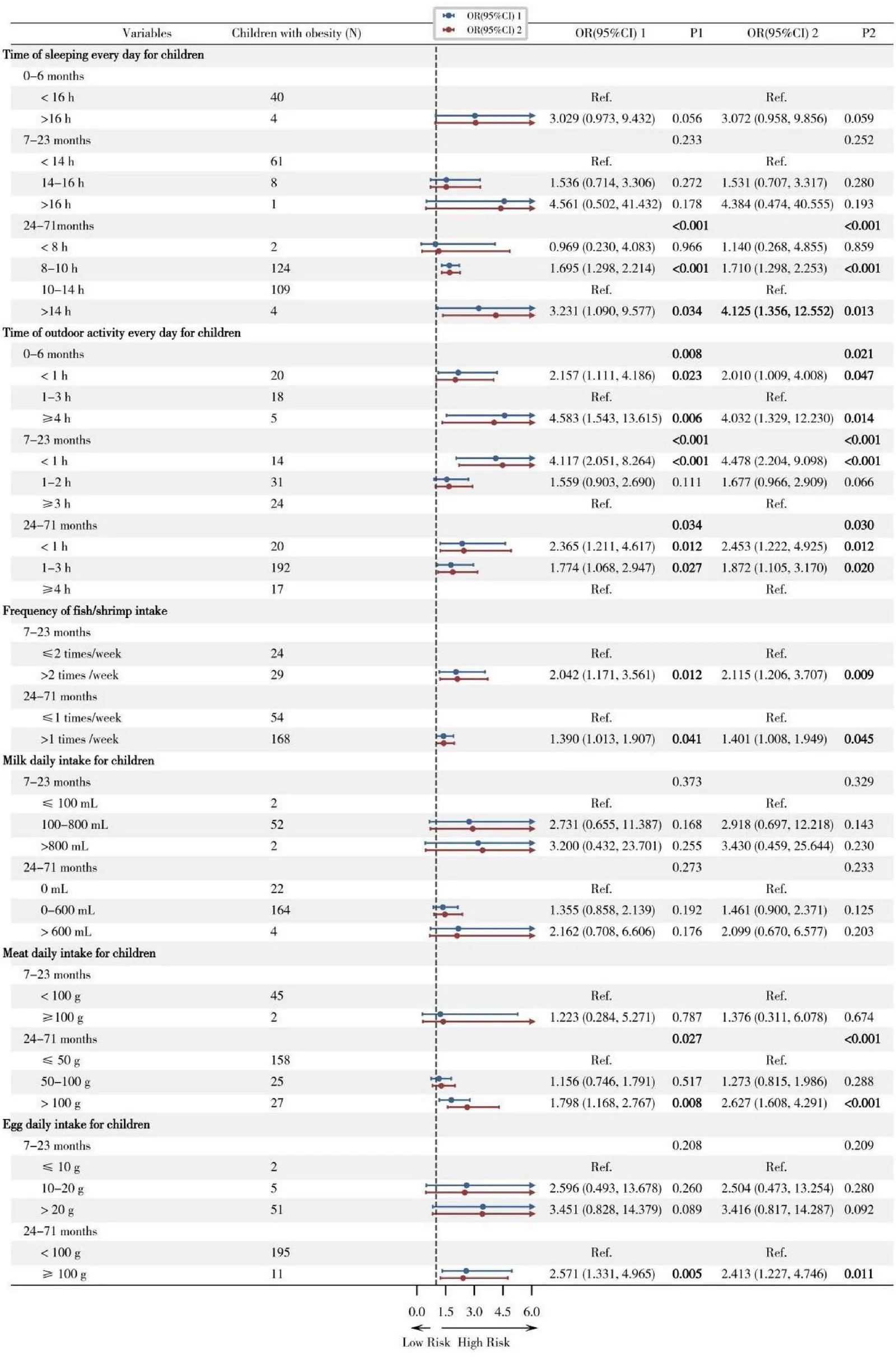
Age-specific associations of lifestyles and dietary factors with obesity in children during the first 6 years of life. P2: Adjusted for sex, age, mode of delivery, birth weight, region and season of investigation.

The age-stratified forest plot (Figure 4) revealed differential associations of lifestyle and dietary factors with childhood obesity before and after adjustment. Sleep duration showed no significant associations across 0–6 months and 7–23 months age groups. But in 24–71 months group, sleep duration was nominally significantly associated with obesity (8–10 h/d: OR (95% CI) = 1.695 (1.298, 2.214) and OR (95% CI) = 1.710 (1.298, 2.253) before and after adjustment; >14 h: OR (95% CI) = 3.231 (1.090, 9.577) and OR (95% CI) = 4.125 (1.356, 12.552) before and after adjustment). Outdoor activity duration was nominally significantly associated with increased obesity risk in infants aged 0–6 months (<1 h/day: OR (95% CI) = 2.157 (1.111, 4.186) and OR (95% CI) = 2.010 (1.009, 4.008) before and after adjustment; ≥ 4 h/day: OR (95% CI) = 4.583 (1.543, 13.615) and OR (95% CI) = 4.032 (1.329, 12.230) before and after adjustment), children aged 7–23 months (<1 h/day: OR (95% CI) = 4.117 (2.051, 8.264) and OR (95% CI) = 4.478 (2.204, 9.098) before and after adjustment), and children aged 24–71 months (<1 h/day: OR (95% CI) = 2.365 (1.211, 4.617) and OR (95% CI) = 2.453 (1.222, 4.925) before and after adjustment; 1–3 h/day: OR (95% CI) = 1.774 (1.068, 2.947) and OR (95% CI) = 1.872 (1.105, 3.170) before and after adjustment). Fish/shrimp intake >2 times/week was nominally significant in children aged 7–23 months [OR (95% CI) = 2.042 (1.171, 3.561) and OR (95% CI) = 2.115 (1.206, 3.707) before and after adjustment), and > 1 time/week in those aged 24–71 months [OR (95% CI) = 1.390 (1.013, 1.907) and OR (95% CI) = 1.401 (1.008, 1.949), before and after adjustment). Milk intake was not significant in any group. Meat intake > 100 g/day was nominally significant only in children aged 24–71 months [OR (95% CI) = 1.798 (1.168, 2.767) and OR (95% CI) = 2.627 (1.608, 4.291) before and after adjustment). Egg intake ≥ 100 g/day was nominally significant in children aged 24–71 months [OR (95% CI) = 2.571 (1.331, 4.965) and OR (95% CI) = 2.413 (1.227, 4.746) before and after adjustment). Findings with confidence intervals close to 1, such as outdoor activity < 1 h/day at 0–6 months (adjusted OR 2.010 (1.009–4.008)] and fish/shrimp intake >1 time/week at 24–71 months (adjusted OR 1.401 (1.008–1.949)], were especially exploratory.

After FDR correction in the adjusted logistic regression models (Supplementary Table 1), the following factors remained significantly associated with the outcome: daily sleep duration >14 h (P2_FDR < 0.001), outdoor activity <1 h/day (vs ≥ 1 h, P2_FDR < 0.001), Preference for vegetarian foods (P2_FDR = 0.005), and daily meat intake >150 g (vs < 50 g, P2_FDR = 0.040).

## Discussion

First, RCS analysis demonstrated a non-linear association between sleep duration and childhood obesity risk. Compared with the reference group (sleep duration 10–14 h), children with sleep duration <10 h and >14 h exhibited elevated obesity prevalence (8.5% and 9.9%, respectively), with corresponding ORs (95% CIs) of 1.327 (1.024, 1.719) and 2.503 (1.640, 3.820) after adjustment. It should be noted that the lower bound of the confidence interval for the former OR lies close to 1.0, indicating limited precision; therefore, this finding was only nominally significant and should be considered should not be exploratory, and this estimate should not to be over-interpreted as a clear risk factor.

This nonlinear sleep duration-obesity relationship has been understudied specifically among children aged 0–6 years (22). A systematic review found linear dose-response only in <10-year-olds or combined overweight and obesity (23). In contrast, a large-scale Jiaxing Birth Cohort (n = 48,922) (24) found sleep duration correlated with childhood overweight/obesity among children aged 3–5 years in a pattern with two risk cutoffs, identifying critical thresholds at <10 h and ≥13 h. Our findings were partially consistent with the short-sleep threshold (<10 h) reported previously but further extend the evidence by identifying >14 h as the upper adverse threshold in children. Additionally, our study recruited participants from 10 cities in Jiangsu Province, which improves regional representativeness compared with single-city sampling, and focused exclusively on the critical early life period (0–6 years).

A Japanese cohort (25,378 children) study indicated that short sleep duration (<11 h/d) at age 2.5 years associated with obesity at 5.5 years in girls (25). The study showed longer nocturnal sleep duration may contribute to abdominal obesity (26). A meta-analysis (19 articles, 0–18 years) reported short sleep increased obesity risk [OR = 1.191), particularly <6 years [OR = 1.304 for <10 h) (26). Consistent with prior physiological studies, insufficient sleep disrupts hormones (lower leptin, higher ghrelin), increasing hunger, and impairs metabolism (including energy balance).

Notably, evidence regarding the adverse impact of excessively long sleep on childhood obesity remains scarce (27, 28), rendering our finding of an increased obesity risk among children sleep duration >14 h novel. In our data set, the >14 h sleep group showed an unadjusted OR of 1.863 (95% CI: 1.269–2.734) and a fully adjusted OR of 2.503 (95% CI: 1.640–3.820) for childhood obesity. The elevated obesity risk observed at sleep duration >14 h is mechanistically puzzling. One speculative explanation is that extremely long time in bed may compress daily physical activity windows and displace active play, thereby reducing total energy expenditure (29). Alternatively, it could reflect underlying chronic low-grade inflammation or circadian misalignment affecting metabolic efficiency (30). However, we emphasize that these speculations lack direct empirical support from the current pediatric data, and the underlying pathways remain largely unexplored in children aged 0–6 years (31). However, childhood obesity is a complex issue that is influenced by a combination of multiple factors. Importantly, reverse causation cannot be ruled out given the cross-sectional design. In children aged 0–6 years, pre-existing obesity or rapid weight gain may predispose to obstructive sleep apnea (OSA) and hypoxia-related daytime drowsiness, which could artificially prolong caregiver-observed sleep duration or time in bed. Thus, the observed association for the >14 h group may partially reflect obesity-driven sleep alterations rather than a causal effect of long sleep on adiposity (32, 33).

Secondly, we found that lack of outdoor activity is an independently associated factor for obesity. The prevalence of obesity among children with outdoor activity time <1 h is 10.7%, significantly higher than in those (6.2%) with outdoor activity time ≥1 h. Compared to children with outdoor activity time ≥1 h, the unadjusted OR (95% CI) for obesity was 1.814 (1.335, 2.464) among children with outdoor activity time <1 h, and the adjusted OR(95% CI) of 2.018 (1.465, 2.779) after adjusting for some factors. This association was further corroborated in age-stratified analyses. Our findings align with prior studies (34, 35). Zhang (34) found that adolescents who did not have ≥1 h of outdoor activity on weekdays were more likely to be overweight/obese and have high waist circumferences from the cross-sectional study in 2,264 adolescents with averaged age of 13.7 years. Chen (35) found that lower frequencies (<3 times/week) or shorter duration (<1 h/time) of outdoor activity from the age of 1–3 years were significantly associated with the presence of obesity from the study of 69,637 mother-child dyads recruited from all kindergartens in the Longhua District of Shenzhen, China. Jiang et al. (36) found that less than 2 h of physical activity per day was one of the 33 higher risks of childhood obesity from the meta-analysis, which incorporated 419 studies focused on obesity.

Thirdly, we also examined how dietary supplements – specifically vitamin D and calcium-impact obesity, focusing on factors like the initial supplementation time, intake in the past 3 months, and daily dosage. We found that the prevalence of obesity was 8.0% who started supplementation <15 days after birth, higher than in those who began 15–42 days. Vitamin D supplementation is recommended for newborns (10 μg per day, up to age 4) (37). But our study indicated that vitamin D supplementation started <15 days after birth will be associated with childhood obesity, with an adjusted OR (95% CI) of 1.686 (1.063, 2.673) compared to those who started vitamin D supplementation 15–42 days. Given that the lower boundary of the confidence interval is close to 1.0, this finding was only nominally significant and should be regarded as exploratory; it indicates a suggestive association rather than a firmly established effect, and the precision of this point estimate should be interpreted with caution. This tentatively suggests a potential optimal window for newborn vitamin D supplementation might lie within 15–42 days of birth, although this observation warrants confirmation in larger cohorts given the wide confidence interval. The systematic review and network meta-analysis (38) noted that extremely high vitamin D doses (>4000 IU/day) may best reduce inflammatory responses and improve insulin resistance in overweight/obese children and adolescents, though the youngest participants in that study were 9 years old. But few studies have focused on vitamin D and obesity in children aged 0–6 years. Our findings preliminarily suggest that the timing of the first vitamin D supplementation in infancy may be associated with obesity in children aged 0–6 years, though the wide confidence interval underscores the need for further validation.

We also investigated calcium supplementation, but no significant associations with obesity were observed, likely due to limited variability in calcium intake patterns among the study participants. Further research with larger, more diverse samples is needed to clarify calcium’s role in early childhood obesity.

The fourth, feeding choices (e.g., formula versus breastfeeding) and timing (e.g., early solid food introduction) were linked to poor dietary habits and subsequent obesity risk (39). Our study found that children who started complementary feeding before 4 months had an obesity prevalence of 9.8% and adjusted OR (95% CI) of 1.665 (1.114, 2.487), consistent with previous research (39, 40). While a study of 8,053 children aged 3–6 years in Jiangsu (41) confirmed breast-feeding’s protective effect against obesity, our research didn’t detect this association, consistent with Shen et al.’s (42) findings. Shen’s research found no significant links between breastfeeding duration, BMI, and fat mass indicators from 2,008 participants aged 3–5 years. These conflicting results highlight the need for more rigorous, longitudinal studies to clarify breast-feeding’s role in preventing obesity in early childhood.

Fifth, we found that children preference for meat had a higher association with obesity. Higher daily meat consumption was associated with a significantly elevated risk of obesity exclusively among children during the first 6 years of life, especially aged 24–71 months. Univariate analysis demonstrated that children daily consuming >150 g of meat exhibited an obesity prevalence of 11.0%, with adjusted OR (95% CI) of 2.627 (1.608, 4.291) compared with those whose daily meat intake was ≤50 g. Children under 6 with meat daily intake > 150 g had an adjusted OR (95% CI) value of 2.060 (1.207, 3.514). However, there is limited research on the relationship between daily meat intake and obesity among children aged 0–6 years (43, 44). Huang et al. (43) found that the intake of meat from livestock, poultry, fish/shrimp among overweight and obese children increased significantly at different ages among 19,529 preschool children aged 3–6 years in Jiashan County of Zhejiang province. Unfortunately, the authors grouped red meat with fish/shrimp in their analysis, making it impossible to determine the meat intake of children aged 3–6. Sarah et al. (44) analyzed five dietary patterns among 2,400 children (average age 8.28 years, 1,597 childhood and 803 adolescence) from six European countries by KIDMED index. However, the authors failed to identify the relationship between meat patterns and obesity in children. Unfortunately, they also didn’t report meat consumption within the dietary patterns.

The sixth, preference for vegetarian foods emerged as a protective factor (as noted in earlier analyses), with adjusted OR (95% CI) of 0.245 (0.107, 0.562), likely due to higher fiber and lower saturated fat intake in plant-based diets. This result was similar to the result in a study from 802 Korean children aged 2–3 years, which showed higher “vegetables & traditional” intake associated with lower obesity risk (45). Segovia-Siapco et al. (46) also noted that vegetarian children are leaner and less prone to cardio-metabolic risks than non-vegetarian peers. In contrast, Laura J. Elliott (47) et al found no association between a vegetarian diet and overweight or obesity in the longitudinal cohort study of 8,907 children (248 vegetarian) aged 6 months to 8 years.

Finally, we examined the effects of fish/shrimp intake frequency, egg intake, and daily milk on childhood obesity. Children with fish/shrimp intake ≥2 times/week were observed to have higher odds compared to those with intake <2 times/week. Stratum-specific obesity counts were 201 out of 2,848 for the ≥2 times/week group versus 79 out of 1488 for the <2 times/week group. The adjusted OR was 1.354 (95% CI: 1.036, 1.771). Given that the lower boundary of this CI was barely above 1.0, this finding was only nominally significant and should be considered exploratory; it suggests a modest positive association that requires cautious interpretation, rather than a robust independent risk effect. Similarly, children with egg daily intake > 60 g had an odds of 2.937 (95% CI: 1.075, 8.024) compared to those without egg daily intake (stratum-specific counts: 23 out of 208 obese in the >60 g group vs. 5 out of 157 in the no-daily-egg group). The corresponding adjusted OR was 2.937 (95% CI: 1.075, 8.024). Although the point estimate is elevated, the confidence interval is extremely wide and its lower limit remains close to the null (1.075), indicating poor statistical precision. Therefore, this result was only nominally significant and should be viewed as as a preliminary hypothesis-generating signal rather than a confirmed risk indicator. What’s more, compared with children who consumed ≤ 100 mL of milk daily, those with daily milk intake ≥600 mL had an OR of 1.830 (95% CI: 1.083, 3.094) [stratum-specific counts: 33 out of 523 (6.3%) vs. 46 out of 839 for the ≤100 mL group]. Since the lower confidence bound (1.083) is only marginally above unity, this association was only nominally significant and should be considered exploratory, and should not be over-interpreted as a clear dose-response effect. Our fish/shrimp finding appears to diverge from a nationwide cohort study (48) that reported a 26% lower risk of abdominal obesity among high seafood consumers aged 6–18 years; this discrepancy may be partly attributable to the much younger age range (0–6 years) in our sample, as well as differences in outcome measures (overall obesity vs. abdominal obesity). Conversely, our egg intake result is consistent with a previous study in preschoolers (43), which also observed higher egg consumption among obese children. Regarding milk, our finding partially aligns with *a prior* cohort study in children aged 4–72 months (49) that cautioned against daily milk intake >500 mL; however, given the very wide and near-null confidence interval in our estimate, this consistency should be interpreted with caution and does not independently confirm a strong causal link.

The strengths of this study are as follows. First,we found a non-linear association between the risk of obesity and sleep duration among children during the first 6 years of life, rather than solely focusing on preschool children. Second, there are few studies on outdoor activities among children aged 0–6 years, and most relevant studies focus on school-age children. Third, this was a large multi-center cross-sectional study. Finally, this study considered the influence of confounding factors like child sex, age, mode of delivery, birth weight, region, and season of investigation.

The present study has several limitations. First, the cross-sectional design precludes causal inference regarding the relationship between associated factors and childhood obesity, and no mechanistic pathways were explored. Second, limited sample sizes within certain subgroups likely reduced the reliability of regression estimates; future studies with larger cohorts are warranted to derive more robust effect estimates. Third, retrospective caregiver-reported data (such as sleep duration) were prone to recall bias. Non-differential misclassification may disproportionately distort the RCS curve at the extreme tail, potentially inflating the observed OR. Thus, the long-sleep association warrants cautious interpretation. Fourth, several well-recognized risk factors for childhood obesity (maternal pre-pregnancy obesity, excessive gestational weight gain, family socioeconomic status) could not be included in the models due to missing data. We selected covariates based on univariate significance, a suboptimal confounding control method that ignores correlations between confounders. Though available maternal indicators showed no univariate association with childhood obesity, unmeasured key variables may still cause residual confounding. Future studies need comprehensive socioeconomic and lifestyle data for sufficient confounding adjustment to verify our results. Fifth, obesity assessment relied solely on ZBMI, subsequent studies should incorporate precise measures of body composition and fat distribution to enable more rigorous analyses. Sixth, our data were collected in 2014–2015, nearly a decade prior to manuscript submission. Over this time-frame, profound shifts have occurred in children’s diets, screen time, outdoor activities and population-level obesity prevalence, which means findings cannot be directly applied to present-day preschool children without careful consideration. While these data serve as a valuable historical baseline amid limited recent large-scale population surveys, cross-temporal extrapolation of our results should be interpreted with great caution. Although core mechanisms hold biological plausibility, we cannot assume the exact same epidemiological relationships would be observed among children today. Seventh, outdoor activity time was assessed as a general behavioral indicator, but specific sun exposure duration, intensity, or the extent of skin exposure were not recorded. Therefore, the variable measured does not directly capture effective sun exposure, which may have led to misclassification of the actual sunlight-related exposure and weakened the precision of our estimates. Finally, without adjusting for total energy intake, we cannot fully distinguish the independent correlation of each food group from the confounding effect of overall dietary energy, and we recommend that future relevant studies include total energy intake for more precise analyses of dietary correlates of childhood obesity.

## Conclusion

This study identified several modifiable lifestyle factors with nominal associated with increased obesity, including non-optimal sleep duration (<10 h or >14 h), limited outdoor activity (<1 h/day), earlier initiation of vitamin D supplementation (<15 days after birth), early introduction of complementary feeding (<4 months), a preference for meat, and high daily consumption of animal-derived products (meat, milk, eggs, and fish/shrimp). A preference for vegetarian foods was identified as an inverse association factor. After FDR correction, longer sleep duration (>14 h), limited outdoor activity (<1 h/day), preference for vegetarian foods, preference for vegetarian foods, and meat intake >150 g/day remained statistically significant. These findings highlight potential targets for preventive strategies and parental education aimed at mitigating the risk of early childhood obesity.

## Data Availability

The raw data supporting the conclusions of this article will be made available by the authors, without undue reservation.

## Supplementary material

Supplementary Table 1 Variable definitions and classification criteria for factors associated with obesity in children under six years old in Jiangsu Province.
