# Supplementary material for: The impact of dietary and lifestyle factors on obesity among chinese children during the first 6 years of life: a 2014-2015 multi-centre study
Source: Front Nutr. 2026 Sep 10;13:1854091. doi: 10.3389/fnut.2026.1854091 (PMC13600868; doi:10.3389/fnut.2026.1854091)
Supplement: Supplementary file 1 [file Table_1.docx]

**Supplementary Table 1 Logistic regression results after FDR correction incorporating all tested exposure variables**

| Variables | N | P1 | P1_FDR |  | P2 | P2_FDR |
| --- | --- | --- | --- | --- | --- | --- |
| Time of sleeping every day for children | 5243 | **<0.001** | **0.003** |  | **<0.001** | **<0.001** |
| < 10 h | 1565 | **<0.001** | **0.003** |  | **0.032** | 0.069 |
| 10-14 h | 3335 |  |  |  |  |  |
| >14 h | 343 | **0.001** | **0.005** |  | **<0.001** | **<0.001** |
| Time of outdoor activity every day for children | 5139 |  |  |  |  |  |
| < 1 h | 505 | **<0.001** | **0.003** |  | **<0.001** | **<0.001** |
| ≥1 h | 4634 |  |  |  |  |  |
| Feeding patterns for infants from birth to 6 months | 5238 | 0.801 | 0.890 |  | 0.873 | 0.925 |
| Breastfeeding | 2700 | 0.607 | 0.759 |  | 0.624 | 0.719 |
| Mixed feeding | 1515 |  |  |  |  |  |
| Artificial feeding | 1023 | 0.917 | 0.983 |  | 0.925 | 0.925 |
| Vitamin D supplementation for infants from birth to 6 months | 5068 |  |  |  |  |  |
| Yes | 3725 |  |  |  |  |  |
| No | 1343 | **0.049** | 0.099 |  | 0.173 | 0.236 |
| The initial time of vitamin D supplementation after birth | 3584 | 0.072 | 0.135 |  | 0.063 | 0.117 |
| <15 days | 351 | **0.031** | 0.066 |  | **0.026** | 0.067 |
| 15-42 days | 1384 |  |  |  |  |  |
| >42 days | 1849 | 0.106 | 0.187 |  | 0.101 | 0.159 |
| The initial time of complementary feeding | 4848 |  |  |  |  |  |
| < 4 months | 306 | **0.015** | **0.041** |  | **0.013** | 0.055 |
| ≥ 4 months | 4542 |  |  |  |  |  |
| Vitamin D supplementation for children in the last 3 months | 5198 |  |  |  |  |  |
| Yes | 1476 | 0.722 | 0.866 |  | 0.087 | 0.146 |
| No | 3722 |  |  |  |  |  |
| Daily dose of vitamin D supplementation for children in the last 3 months | 1303 |  |  |  |  |  |
| ≤400 IU | 898 | 0.961 | 0.987 |  | 0.788 | 0.876 |
| >400 IU | 405 |  |  |  |  |  |
| Calcium supplementation for children in the last 3 months | 5151 |  |  |  |  |  |
| Yes | 985 |  |  |  |  |  |
| No | 4166 | 0.531 | 0.711 |  | 0.523 | 0.628 |
| Daily dose of calcium supplementation for children | 782 |  |  |  |  |  |
| ≤300 mg | 734 | 0.118 | 0.191 |  | 0.127 | 0.181 |
| >300 mg | 48 |  |  |  |  |  |
| Dietary habit | 4194 | **< 0.001** | **0.003** |  | **< 0.001** | **< 0.001** |
| No preference | 2534 |  |  |  |  |  |
| Preference for meat | 887 | **<0.001** | **0.003** |  | **0.022** | 0.067 |
| Preference for vegetarian foods | 342 | **0.002** | **0.009** |  | **0.001** | **0.005** |
| Preference for sweets | 431 | 0.220 | 0.330 |  | 0.111 | 0.167 |
| Frequency of fish/shrimp intake | 4336 |  |  |  |  |  |
| < 2 times/week | 1488 |  |  |  |  |  |
| ≥2 times /week | 2848 | **0.027** | 0.062 |  | **0.032** | 0.069 |
| Milk daily intake for children | 4492 | 0.783 | 0.890 |  | 0.078 | 0.137 |
| ≤ 100 ml | 839 |  |  |  |  |  |
| 0-600 ml | 3130 | 0.545 | 0.711 |  | 0.221 | 0.288 |
| ≥ 600 ml | 523 | 0.526 | 0.711 |  | **0.024** | 0.067 |
| Meat daily intake for children | 4023 | **0.027** | 0.062 |  | **0.024** | 0.067 |
| < 50 g | 3008 |  |  |  |  |  |
| 50-150 g | 818 | 0.987 | 0.987 |  | 0.895 | 0.925 |
| > 150 g | 197 | **0.008** | **0.027** |  | **0.008** | **0.040** |
| Egg daily intake for children | 4216 | **0.007** | **0.026** |  | **0.027** | 0.067 |
| 0 g | 157 |  |  |  |  |  |
| 0-60 g | 3851 | 0.121 | 0.191 |  | 0.273 | 0.342 |
| >60 g | 208 | **0.009** | **0.027** |  | **0.036** | 0.071 |

Notes: P1: crude P value; P1_FDR, FDR-adjusted P corresponding to P1; P2, P value after adjustment; P2_FDR, FDR-adjusted P corresponding to P2.
